# Supplementary material for: Thermal and Mechanical Characterization of Yarn Samples from Flemish Tapestry of the Sixteenth Century
Source: Molecules. 2022 Dec 2;27(23):8450. doi: 10.3390/molecules27238450 (PMC9737756; doi:10.3390/molecules27238450)
Supplement: Supplementary file 1 [file molecules-27-08450-s001.zip › molecules-2043577-SI.pdf]

Thermal and Mechanical Characterization of Yarn samples from Flemish Tapestry of the Sixteenth Century

Maria Rita Caruso [1], Lorenzo Lisuzzo [1], Giuseppe Cavallaro [*,1], Giacomo Mirto [2] Stefana Milioto [1] and Giuseppe Lazzara[1]

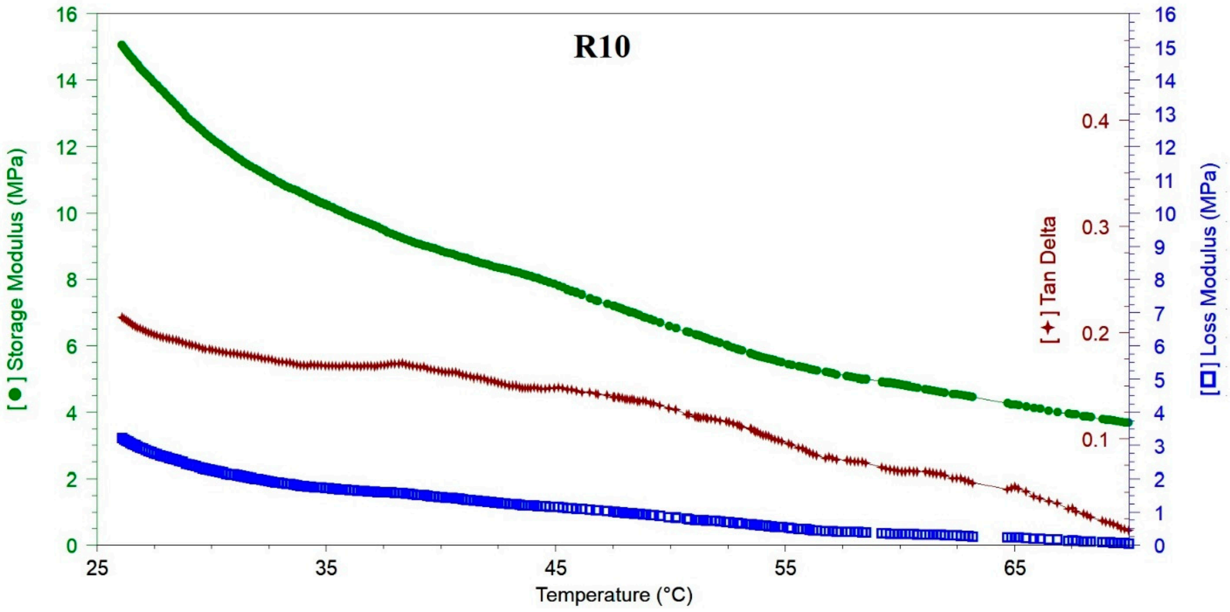

Figure S1. Effect of temperature on the rheological properties (storage modulus, loss modulus and tan (δ)) of the white warp yarn.

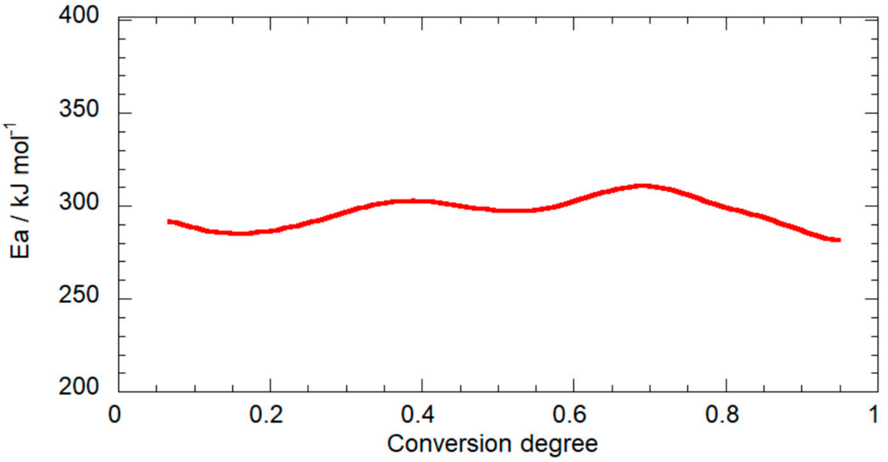

Figure S2. Activation energy as a function of the conversion degree obtained from MTG for silk sample

Table S1. Water uptake results at 25 ° C and relative humidity of 75%.

	Water uptake % **After 15 days**	Water uptake % **After 30 days**
Merino wool	6.5	6.4
Blue warp	9.3	9.5
Red warp	19.1	19.4
Green warp	21.1	21.3
Brown warp	18.0	17.9
“Botto” Silk	16.3	16.7
Yellow weft	35.4	35.2
